# Supplementary material for: A degradative to secretory autophagy switch mediates mitochondria clearance in the absence of the mATG8-conjugation machinery
Source: Nat Commun. 2022 Jun 28;13:3720. doi: 10.1038/s41467-022-31213-7 (PMC9240011; doi:10.1038/s41467-022-31213-7)
Supplement: Supplementary file 1 — Supplementary Information [file 41467_2022_31213_MOESM1_ESM.pdf]

Supplementary Figures

A degradative to secretory autophagy switch mediates mitochondria clearance in the absence of the mATG8-conjugation machinery

Hayden Weng Siong Tan[1,2], Guang Lu[1,3], Han Dong[1], Yik-Lam Cho[1], Auginia Natalia[4], Liming Wang[1,5], Charlene Chan[6], Dennis Kappei[6,7,8], Reshma Taneja[1,2], Shuo-Chien Ling[1], Huilin Shao[4], Shih-Yin Tsai[1], Wen-Xing Ding[9], Han-Ming Shen[1,10,*]

[1] Department of Physiology, Yong Loo Lin School of Medicine, National University of Singapore, Singapore, Singapore
[2] NUS Graduate School (Integrative Sciences and Engineering Programme), National University of Singapore, Singapore, Singapore
[3] Zhongshan School of Medicine, Sun Yat-sen University, Guangzhou, China
[4] Institute for Health Innovation & Technology, National University of Singapore, Singapore, Singapore
[5] School of Biomedical Sciences, Hunan University, Changsha, China
[6] Cancer Science Institute of Singapore, National University of Singapore, Singapore, Singapore
[7] Department of Biochemistry, Yong Loo Lin School of Medicine, National University of Singapore, Singapore, Singapore
[8] NUS Center for Cancer Research, Yong Loo Lin School of Medicine, National University of Singapore, Singapore, Singapore
[9] Department of Pharmacology, Toxicology and Therapeutics, The University of Kansas Medical Center, Kansas City, KS, USA
[10] Faculty of Health Sciences, University of Macau, Macau, China
[*] Corresponding author: hmshen@um.edu.mo

Suppl Fig 1

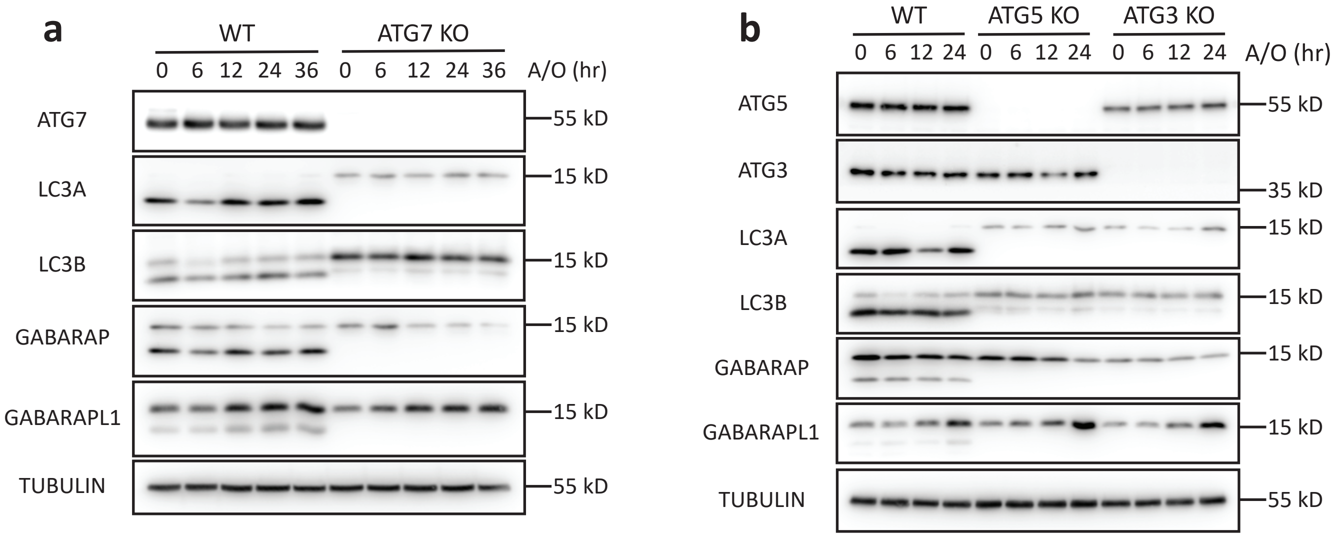

Supplementary Fig. 1 Characterization of mATG8-conjugation system knockouts.

a Validation of ATG7 knockout in HeLa stably expressing mCherry-Parkin. **b** Validation of ATG5 and ATG3 knockout in HeLa stably expressing mCherry-Parkin.

Suppl Fig 2

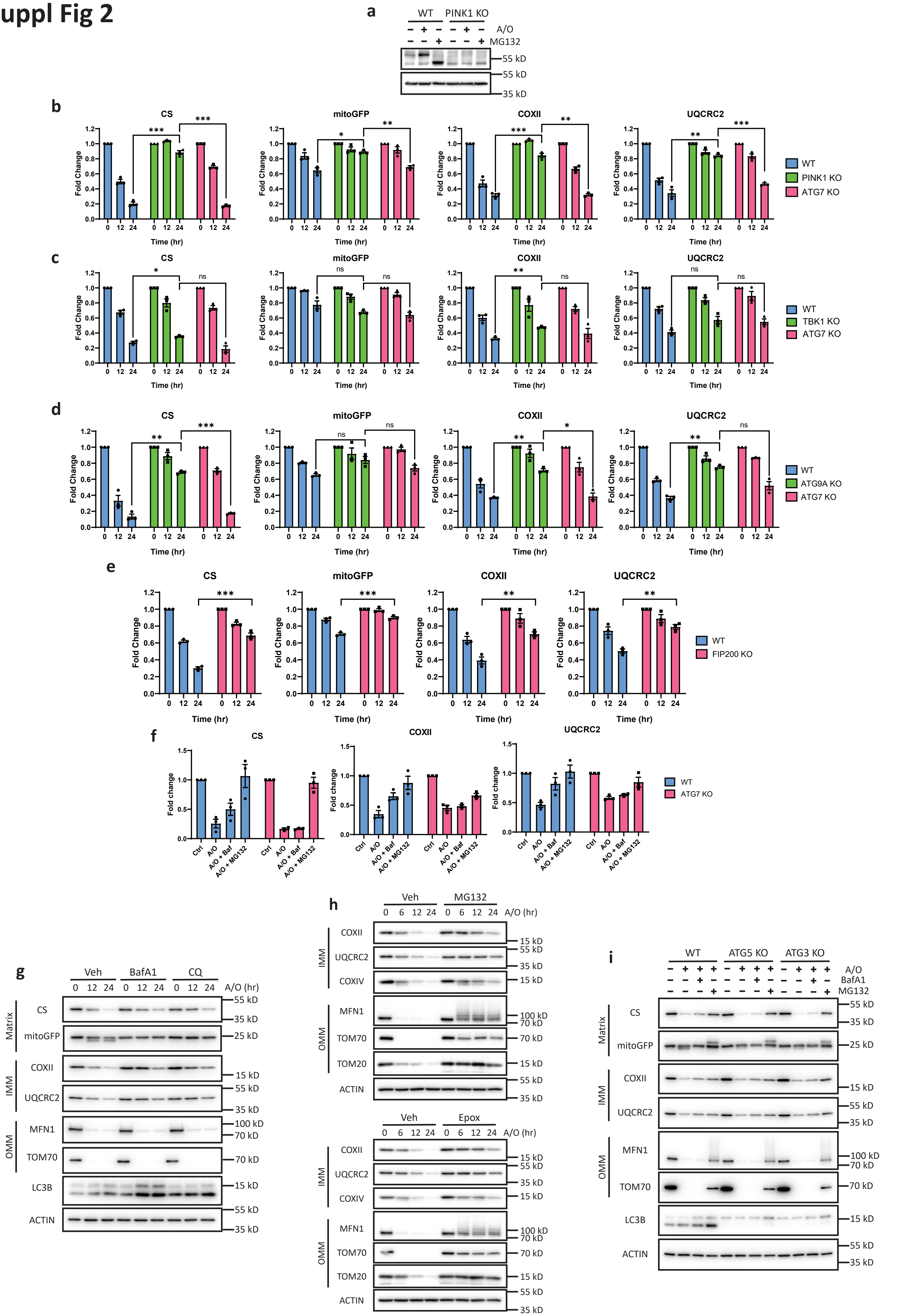
a
WT
PINK1 KO
A/O
MG132
55 kD
35 kD
b
CS
mitoGFP
COXII
UQCRC2
Fold Change
Time (hr)
WT
PINK1 KO
ATG7 KO
c
TBK1 KO
d
ATG9A KO
e
FIP200 KO
f
Fold change
Ctrl
A/O
A/O + Baf
A/O + MG132
g
Veh
BafA1
CQ
A/O (hr)
Matrix
IMM
OMM
MFN1
TOM70
LC3B
ACTIN
100 kD
70 kD
25 kD
15 kD
h
MG132
COXIV
TOM20
Epox
i
ATG5 KO
ATG3 KO

Supplementary Fig. 2 Effect of lysosomal or proteasomal inhibition on mitochondria clearance and protein quantifications.

a WT and PINK1-KO cells were treated with A/O or MG132 for 4 hrs to accumulate full length or cleaved PINK1 respectively. **b** Quantification of mitochondrial protein changes from WT, PINK1-KO and ATG7-KO cells in Fig. 2a. **c** Quantification of mitochondrial protein changes from WT, TBK1-KO and ATG7-KO cells in Fig. 2b. **d** Quantification of mitochondrial protein changes from WT, ATG9A-KO and ATG7-KO cells in Fig. 2c. Mean of n = 3 independent replicates ± SEM are shown for (**b**-**d**). P values are calculated by two-way ANOVA followed by Dunnett's multiple comparisons test. *P < 0.05, **P < 0.01, ***P < 0.001, and ns denotes not significant. See source data for exact P values. **e** Quantification of mitochondrial protein changes from WT and FIP200-KO cells in Fig. 2d. Mean of n = 3 independent replicates ± SEM is shown. P values are calculated by two-tailed student's T test. *P < 0.05, **P < 0.01, ***P < 0.001, and ns denotes not significant. See source data for exact P values. **f** Quantification of mitochondrial protein changes from WT and ATG7-KO cells in Fig. 2f. Mean of n = 3 independent replicates ± SEM is shown. **g** HeLa stably expressing mCherry-Parkin was treated with A/O in the absence or presence of Bafilomycin A1 (BafA1, 200 nM) or chloroquine (CQ, 100 µM). **h** HeLa stably expressing YFP-Parkin were treated with A/O for the indicated duration. Proteasomal degradation was inhibited by MG132 (10 µM) or epoxomicin (1 µM). **i** WT, ATG5-KO and ATG3-KO cells stably expressing mCherry-Parkin were treated with A/O for 24 hrs, with or without Bafilomycin A1 (BafA1, 200 nM) or MG132 (10µM) to inhibit lysosomal or proteasome degradation respectively.

Suppl Fig 3

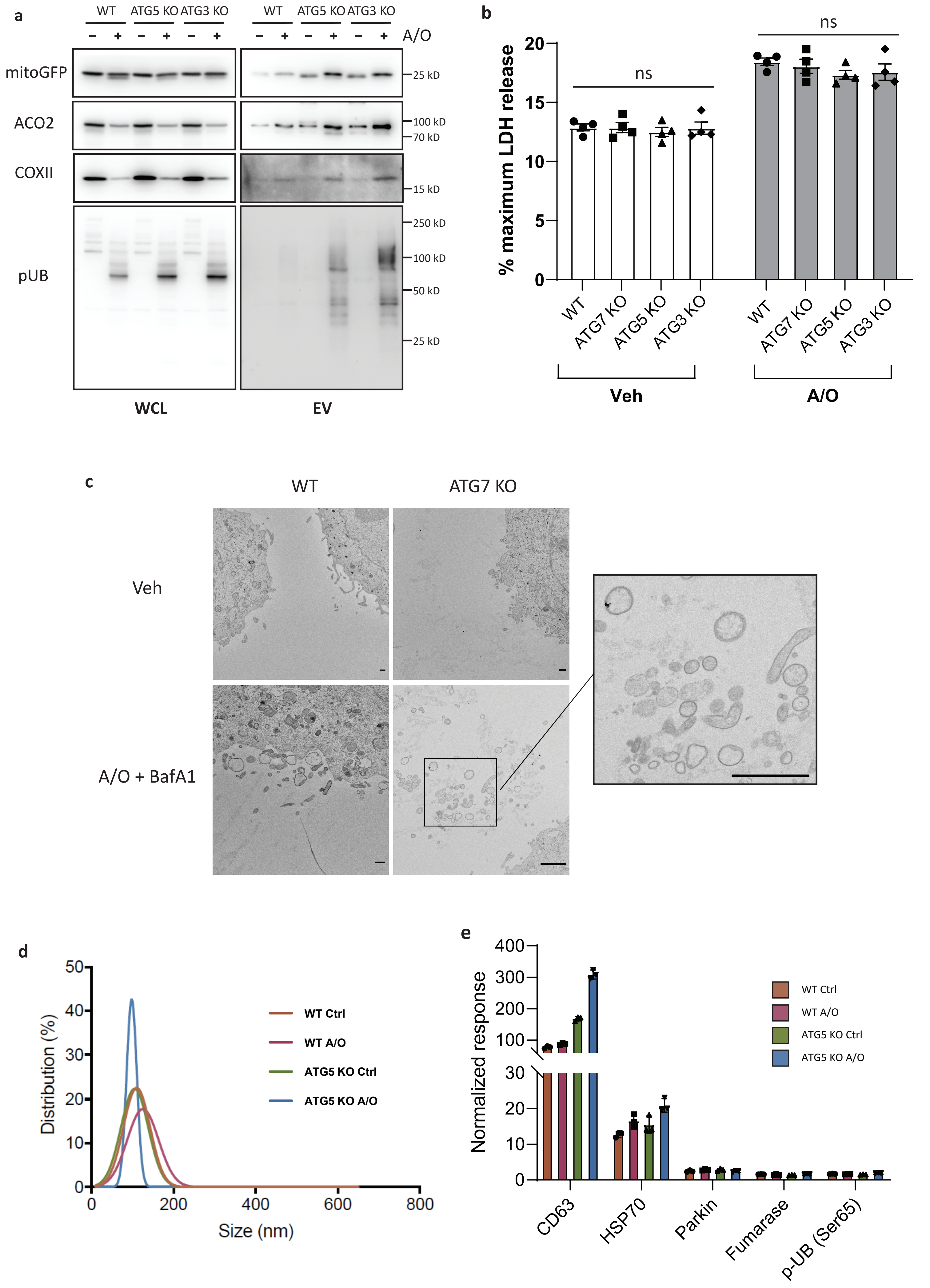

a
WT
ATG5 KO
ATG3 KO
A/O
mitoGFP
ACO2
COXII
pUB
25 kD
100 kD
70 kD
15 kD
250 kD
50 kD
WCL
EV
b
% maximum LDH release
ns
WT
ATG7 KO
ATG5 KO
ATG3 KO
Veh
A/O
c
WT
ATG7 KO
Veh
A/O + BafA1
d
Distribution (%)
Size (nm)
WT Ctrl
WT A/O
ATG5 KO Ctrl
ATG5 KO A/O
e
Normalized response
CD63
HSP70
Parkin
Fumarase
p-UB (Ser65)

Supplementary Fig. 3 Characterization of secreted mitochondria in other mATG8-conjugation defective cell lines.

a Conditioned media from WT, ATG5-KO, ATG3-KO HeLa stably expressing mCherry-Parkin treated with A/O for 24 hr were collected. Extracellular vesicles (EVs) were isolated by differential ultracentrifugation and immunoblotted for mitochondria markers. **b** LDH release assay from WT, ATG7-KO, ATG5-KO and ATG3-KO cells treated with or without A/O for 24 hrs. Mean of n = 4 independent replicates ± SEM is shown. P values are calculated by two-way ANOVA followed by Dunnett's multiple comparisons test. *P < 0.05, **P < 0.01, ***P < 0.001, and ns denotes not significant. See source data for exact P values. **c** Representative TEM images of extracellular regions from WT or ATG7-KO HeLa stably expressing mCherry-Parkin untreated or treated with A/O + BafA1. Scale bar represents 500 nm. **d** Nanoparticle tracking analysis of EVs from WT and ATG5-KO cells untreated or treated with A/O for 24 hrs. **e** nPLEX analysis of mitochondria cargo from immunocaptured CD63+ EVs isolated from WT and ATG5-KO cells stably expressing YFP-Parkin untreated or treated with A/O for 24 hrs. Mean of n = 3 technical replicates ± SD is shown.

Suppl Fig 4

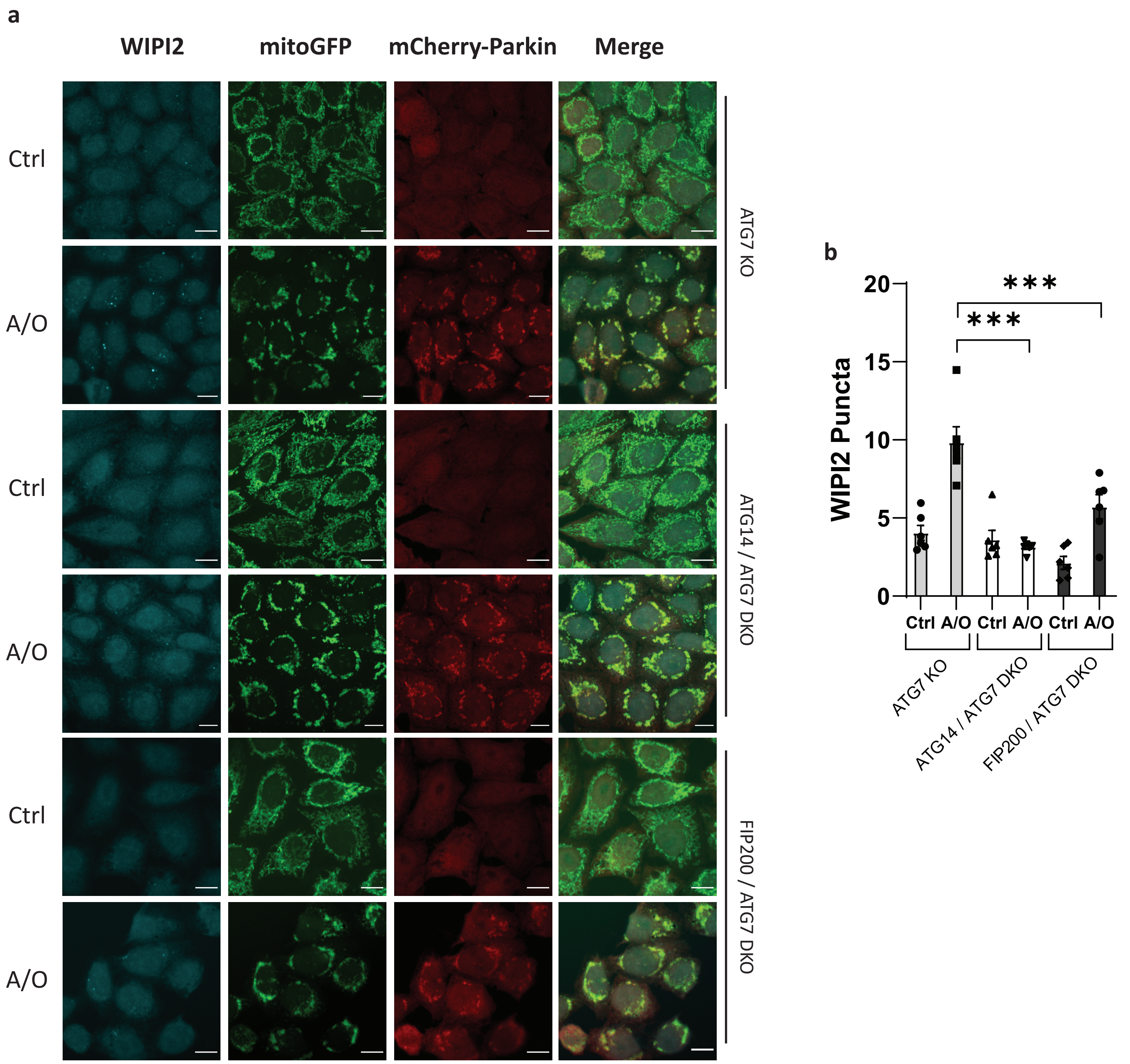

Supplementary Fig. 4 Mitophagosome formation in ATG14/ATG7 and FIP200/ATG7 DKO cells.

a Immunostaining of WIPI2 (cyan), mito-GFP (green) and mCherry-Parkin (red) in ATG7-KO, ATG14/ATG7 DKO and FIP200/ATG7 DKO cells treated with A/O for 2.5 hr. Scale bar = 10 µm. **b** Quantification of WIPI2 puncta are shown. Mean of n = 6 fields of view ± SEM is shown. P values are calculated by two-way ANOVA followed by Dunnett's multiple comparisons test. *P < 0.05, **P < 0.01, ***P < 0.001, and ns denotes not significant. See source data for exact P values.

Suppl Fig 5

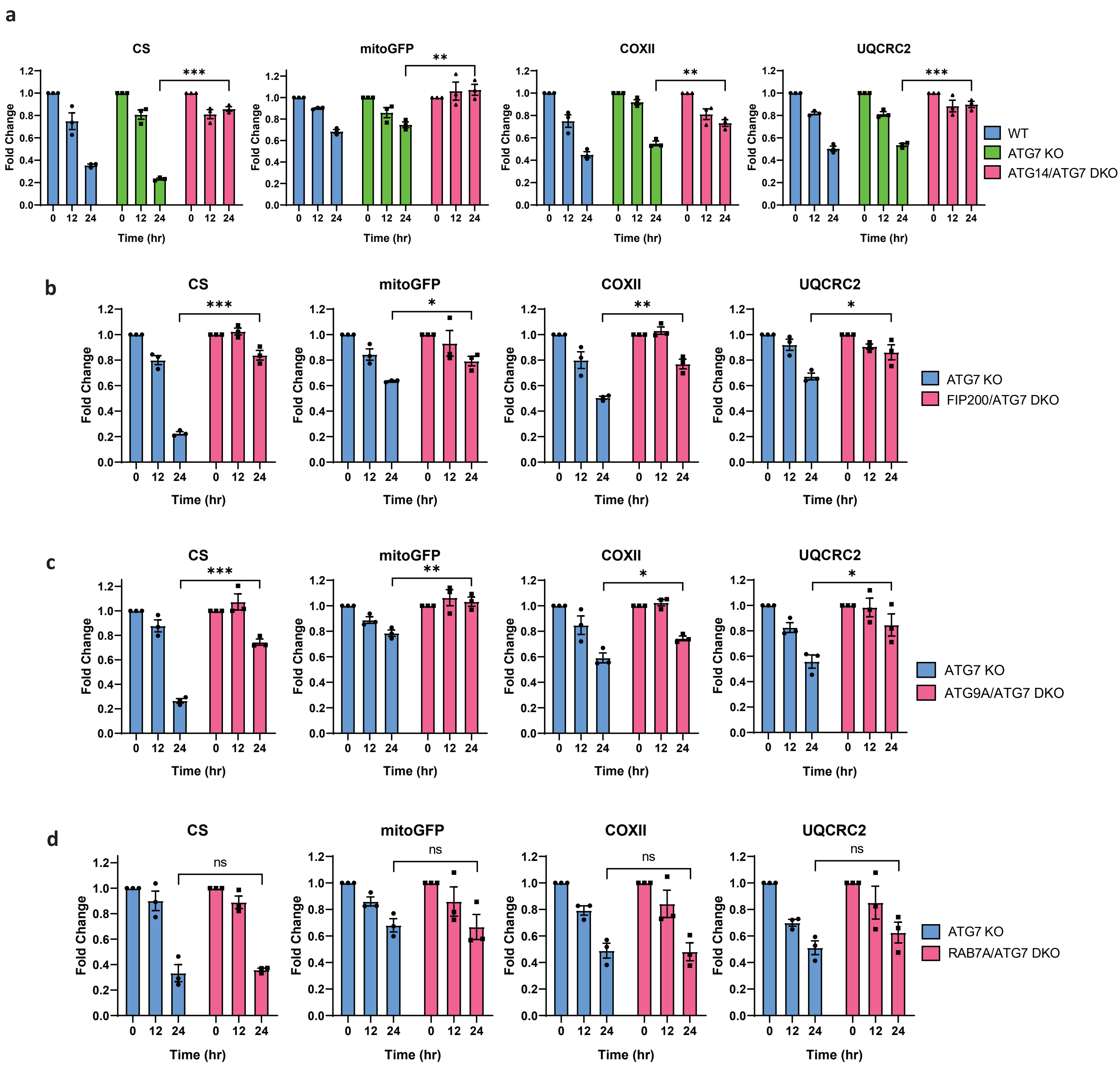

Supplementary Fig. 5 Quantifications of mitochondria protein changes.

a Quantification of mitochondrial protein changes in WT, ATG7-KO and ATG14/ATG7 DKO cells. **b** Quantification of mitochondrial protein changes in ATG7-KO and FIP200/ATG7 DKO cells. **c** Quantification of mitochondrial protein changes in in ATG7-KO and ATG9A/ATG7 DKO cells. **d** Quantification of mitochondrial protein changes in in ATG7-KO and RAB7A/ATG7 DKO cells. Mean of n = 3 independent replicates ± SEM are shown for (**a**-**d**). P values are calculated by two-tailed student's T test. *P < 0.05, **P < 0.01, ***P < 0.001, and ns denotes not significant. See source data for exact P values.
