# Supplementary material for: A degradative to secretory autophagy switch mediates mitochondria clearance in the absence of the mATG8-conjugation machinery
Source: Nat Commun. 2022 Jun 28;13:3720. doi: 10.1038/s41467-022-31213-7 (PMC9240011; doi:10.1038/s41467-022-31213-7)
Supplement: Supplementary file 3 — Description of Additional Supplementary Files [file 41467_2022_31213_MOESM3_ESM.pdf]

File name: Supplementary Data 1

Description: Proteins identified by SILAC proteomics of extracellular vesicles.

File name: Supplementary Data 2

Description: List of CRISPR gRNAs and qPCR primers used in study.
