# Supplementary material for: A degradative to secretory autophagy switch mediates mitochondria clearance in the absence of the mATG8-conjugation machinery
Source: Nat Commun. 2022 Jun 28;13:3720. doi: 10.1038/s41467-022-31213-7 (PMC9240011; doi:10.1038/s41467-022-31213-7)
Supplement: Supplementary file 6 — Reporting Summary [file 41467_2022_31213_MOESM6_ESM.pdf]

nature portfolio

Corresponding author(s): Han-Ming Shen

Last updated by author(s): May 23, 2022

Reporting Summary

Nature Portfolio wishes to improve the reproducibility of the work that we publish. This form provides structure for consistency and transparency in reporting. For further information on Nature Portfolio policies, see our Editorial Policies and the Editorial Policy Checklist.

Statistics

For all statistical analyses, confirm that the following items are present in the figure legend, table legend, main text, or Methods section.

n/a	Confirmed	
☐	☒	The exact sample size (*n*) for each experimental group/condition, given as a discrete number and unit of measurement
☐	☒	A statement on whether measurements were taken from distinct samples or whether the same sample was measured repeatedly
☐	☒	The statistical test(s) used AND whether they are one- or two-sided<br>*Only common tests should be described solely by name; describe more complex techniques in the Methods section.*
☒	☐	A description of all covariates tested
☒	☐	A description of any assumptions or corrections, such as tests of normality and adjustment for multiple comparisons
☐	☒	A full description of the statistical parameters including central tendency (e.g. means) or other basic estimates (e.g. regression coefficient) AND variation (e.g. standard deviation) or associated estimates of uncertainty (e.g. confidence intervals)
☐	☒	For null hypothesis testing, the test statistic (e.g. *F*, *t*, *r*) with confidence intervals, effect sizes, degrees of freedom and *P* value noted<br>*Give P values as exact values whenever suitable.*
☒	☐	For Bayesian analysis, information on the choice of priors and Markov chain Monte Carlo settings
☒	☐	For hierarchical and complex designs, identification of the appropriate level for tests and full reporting of outcomes
☒	☐	Estimates of effect sizes (e.g. Cohen's *d*, Pearson's *r*), indicating how they were calculated

Our web collection on statistics for biologists contains articles on many of the points above.

Software and code

Policy information about availability of computer code

Data collection	ZEN Black (Zeiss, 2011)<br>FV31-ASW (Olympus, V2.1.1.98)<br>NS300 (Nanosight, v3.3)<br>MaxQuant (v1.5.2.8)<br>CFX Manager (Biorad, v3.0)<br>Image Quant LAS500 (GE Life Sciences, v1.1.0)
Data analysis	FIJI (ImageJ, v1.53)<br>Prism (Graphpad, v8.0.2)<br>OriginPro (OriginLab, 2019b)<br>Microsoft excel (Microsoft, 2016)<br>Imaris (Oxford Instruments, v7.4)

For manuscripts utilizing custom algorithms or software that are central to the research but not yet described in published literature, software must be made available to editors and reviewers. We strongly encourage code deposition in a community repository (e.g. GitHub). See the Nature Portfolio guidelines for submitting code & software for further information.

Data

Policy information about availability of data

All manuscripts must include a data availability statement. This statement should provide the following information, where applicable:

- Accession codes, unique identifiers, or web links for publicly available datasets
- A description of any restrictions on data availability
- For clinical datasets or third party data, please ensure that the statement adheres to our policy

Data supporting this study are provided within the paper and supplementary files. The mass spectrometry data generated from this study has been deposited to the ProteomeXchange Consortium via PRIDE partner repository with the dataset identifier PXD025356. Source data are provided with this paper.

Field-specific reporting

Please select the one below that is the best fit for your research. If you are not sure, read the appropriate sections before making your selection.

☒ Life sciences ☐ Behavioural & social sciences ☐ Ecological, evolutionary & environmental sciences

For a reference copy of the document with all sections, see nature.com/documents/nr-reporting-summary-flat.pdf

Life sciences study design

All studies must disclose on these points even when the disclosure is negative.

Sample size	No sample size calculation was used to predetermine the sample size. The number of independent biological replicates for cell based experiments (stated in the Figure legends) were based on experience from similar experiments in our previously published studies and consistent with the current practices in the field. The number of animals used for mice experiments are stated in the Figure legends and were determined based on experience from our previous animal studies to estimate the sample size required to observe and quantify phenotype changes.
Data exclusions	No data were excluded from analysis
Replication	All experiments were repeated to ensure reproducibility. The number of experimental replicates are stated in the Figure legends.
Randomization	For cell based studies, randomization was irrelevant as cells for each experiment were processed and analyzed in parallel. For animal experiments, WT or ATG7-mKO mice were randomly assigned to rest or exercise groups
Blinding	Operators were blinded to the mouse genotype during exercise training and tissue collection. For cell based immunoblotting experiments, operators were not blinded to the experimental groups during collection and analysis as the order of samples was required for data generation. For immunoflourescence based experiments, operators were not blinded as random image fields were taken for each group and quantified using the same software parameters to eliminate bias.

Reporting for specific materials, systems and methods

We require information from authors about some types of materials, experimental systems and methods used in many studies. Here, indicate whether each material, system or method listed is relevant to your study. If you are not sure if a list item applies to your research, read the appropriate section before selecting a response.

Materials & experimental systems

n/a	Involved in the study	
☐	☒	Antibodies
☐	☒	Eukaryotic cell lines
☒	☐	Palaeontology and archaeology
☐	☒	Animals and other organisms
☒	☐	Human research participants
☒	☐	Clinical data
☒	☐	Dual use research of concern

Methods

n/a	Involved in the study	
☒	☐	ChIP-seq
☒	☐	Flow cytometry
☒	☐	MRI-based neuroimaging

Antibodies

Antibodies used	Primary antibodies and dilutions<br><br>Citrate synthase (Cell Signalling Technology, #14309, WB:1/1000)<br>GFP (Cell Signalling Technology, #2956, WB:1/3000)

mitofusin-1 (Cell Signalling Technology, #14739, WB:1/1000)
Atg7 (Cell Signalling Technology, #8558, WB:1/1000)
Atg5 (Cell Signalling Technology, #12994, WB:1/1000)
Atg3 (Cell Signalling Technology, #3415, WB:1/1000)
Atg14 (Cell Signalling Technology, #5504, WB:1/1000)
TBK1/NAK (Cell Signalling Technology, #38066, WB:1/1000)
PINK1 (Cell Signalling Technology, #6946, WB:1/1000)
FIP200 (Cell Signalling Technology, #12436, WB:1/1000)
LC3A (Cell Signalling Technology, #4599, WB:1/1000)
GABARAP (Cell Signalling Technology, #13733, WB:1/1000)
GABARAPL1 (Cell Signalling Technology, #26632, WB:1/1000)
HSP60 (Cell Signalling Technology, #12165, WB:1/1000, IF:1/500)
Tom20 (Cell Signalling Technology, #42406, WB:1/1000)
Aco2 (Cell Signalling Technology, #6571, WB:1/1000)
SDHA (Cell Signalling Technology, #11998, WB:1/1000)
CD9 (Cell Signalling Technology, #13403, WB:1/1000)
Rab7 (Cell Signalling Technology, #9367, WB:1/1000)
phospho-TBK1/NAK (Ser172) (Cell Signalling Technology, #5483, WB:1/1000)
phospho-STING (Ser366) (Cell Signalling Technology, #50907, WB:1/1000)
STING (Cell Signalling Technology, #13647, WB:1/1000)
phospho-IRF-3 (Ser386) (Cell Signalling Technology, #37829, WB:1/1000)
IRF-3 (Cell Signalling Technology, #11904, WB:1/1000)
MTCO2 [COXII] (Abcam, ab110258, WB:1/1000)
UQCRC2 (Abcam, ab203832, WB:1/3000)
Atg9a (Abcam, ab108338, WB:1/1000)
CD63 (Abcam, ab59479, WB:1/1000)
NDP52 (Abcam, ab68588, WB:1/1000)
Tom70 (Santa Cruz Biotechnology, sc-390545, WB:1/1000)
LAMP2 (Santa Cruz Biotechnology, sc-18822, IF:1/200)
LC3B (Sigma-Aldrich, L7543, WB:1/3000),
Tubulin (Sigma-Aldrich, T5168, WB:1/5000)
Actin (Sigma-Aldrich, A5441, WB:1/5000)
phospho-ubiquitin (Ser65) (Merck Millipore, abs1513; WB:1/1000)
Atg18 [WIPI2] (Merck Millipore, MABC91, IF:1/200)
Anti-DNA (Progen Biotechnik, 61014, IF:1/200)
Anti-Fumarase (Novus Biologicals, NBP2-59442)
Anti-Parkin (Novus Biologicals, H00005071-M01)
Biotin anti-human IgG Fc Recombinant Antibody (Biolegend, 366918)

Secondary antibodies:

HRP-linked anti-mouse IgG (Cell Signalling Technology, #7076, WB:1/2000)
HRP-linked anti-rabbit IgG (Cell Signalling Technology, #7074, WB:1/2000)
Goat anti-Mouse IgG (H+L) Cross-Adsorbed Secondary Antibody, Alexa Fluor™ 405 (ThermoFisher, #A-31553, IF:1/200)
Goat anti-Rabbit IgG (H+L) Cross-Adsorbed Secondary Antibody, Alexa Fluor™ 633 (ThermoFisher, #A-21070, IF:1/200)
Goat anti-Mouse IgG (H+L) Cross-Adsorbed Secondary Antibody, Alexa Fluor™ 555 (ThermoFisher, #A-21422, IF:1/200)

Validation

Antibodies used in this study are commercially available and validated by the companies. Validation information for each antibody can be found on the manufacturer's website.

Eukaryotic cell lines

Policy information about cell lines

Cell line source(s)

HeLa and HEK293T cells were obtained from ATCC.

Authentication

Cell lines used in this study were not authenticated by us.

Mycoplasma contamination

Cell lines used in this study were tested negative for mycoplasma contamination.

Commonly misidentified lines
(See ICLAC register)

No commonly misidentified cell lines were used in this study.

Animals and other organisms

Policy information about studies involving animals; ARRIVE guidelines recommended for reporting animal research

Laboratory animals

Muscle specific ATG7 knockout mice were generated by crossing Atg7-floxed (Atg7f/f) mice with transgenic mice bearing the Cre recombinase gene driven by the muscle creatine kinase promoter (Ckmm-cre). Atg7 flox mice (Atg7f/f) were generated and provided by the RIKEN BRC through the National Bio-Resource Project of the MEXT (Japan). Ckmm-cre mice were purchased from The Jackson Laboratory (USA). All mice used in this study were backcrossed to the C57BL/6J background for 7 generations. Mice were housed in a temperature-controlled environment (20-24°C temperature; 40-60% humidity) with a 12 hr light/dark cycle and had access to food

March 2021

	and water ad libitum. 4 month old male mice were used in this study.
Wild animals	No wild animals were used in this study.
Field-collected samples	No field samples were collected in this study.
Ethics oversight	All animal studies were reviewed and approved by the NUS Institutional Animal Care and Use Committee (IACUC; R17-0195).

Note that full information on the approval of the study protocol must also be provided in the manuscript.
